# Emotion Regulation as a Time-Invariant and Time-Varying Covariate Predicts Outcome in an Internet-Based Psychodynamic Treatment Targeting Adolescent Depression

**DOI:** 10.3389/fpsyt.2020.00671

**Published:** 2020-07-14

**Authors:** Jakob Mechler, Karin Lindqvist, Fredrik Falkenström, Per Carlbring, Gerhard Andersson, Björn Philips

**Affiliations:** ^1^ Department of Psychology, Stockholm University, Stockholm, Sweden; ^2^ Department of Behavioural Sciences and Learning, Linköping University, Linköping, Sweden; ^3^ Department of Clinical Neuroscience, Karolinska Institute, Stockholm, Sweden

**Keywords:** emotion regulation, internet-based treatment, psychodynamic, psychotherapy process, adolescents, depression, mechanism of change

## Abstract

**Objective:**

Although psychodynamic psychotherapy is efficacious in the treatment of depression, research on mechanisms of change is still scarce. The aim of this study was to investigate if and how emotion regulation affects outcome both as a time-invariant and a lagged time-varying predictor.

**Method:**

The sample consisted of 67 adolescents diagnosed with major depressive disorder, attending affect-focused psychodynamic internet-based treatment (IPDT). Linear mixed models were used to analyze emotion regulation as a baseline predictor and to assess the effect of within-person changes in emotion regulation on depression.

**Results:**

Analyses suggested that emotion regulation at baseline was a significant predictor of outcome, where participants with relatively larger emotion regulation deficits gained more from IPDT. Further, the results showed a significant effect of improved emotion regulation on subsequent depressive symptomatology. When not controlling for time, increased emotion regulation explained 41.23% of the variance in subsequent symptoms of depression. When detrending the results were still significant, but the amount of explained variance was reduced to 8.7%.

**Conclusion:**

The findings suggest that patients with relatively larger deficits in emotion regulation gain more from IPDT. Decreased emotion regulation deficits seem to act as a mechanism of change in IPDT as it drives subsequent changes in depression.

**Clinical Trial Registration**

International Standard Randomised Controlled Trial Number (ISRCTN) 16206254, https://doi.org/10.1186/ISRCTN16206254.

## Introduction

Depression is the fourth leading cause of illness and disability among young people aged 15–19 years (1). Depression rates increase dramatically from childhood to adolescence (2), and many individuals will not receive any treatment for their condition (3, 4). Accordingly, there is a pressing need for accessible as well as cost- and time-efficient treatments for adolescent depression. One recent response to this need is the development and evaluation of internet-based treatments, which have the potential to reach and treat a larger number of patients (5). Internet-based cognitive behavioral therapy (ICBT) generally seems to perform on par with traditional face-to-face treatment delivered individually or in group (6), but to our knowledge no such head-to-head comparisons exist in the treatment of adolescent psychopathology. There are no studies where internet-based psychodynamic psychotherapy (IPDT) is compared to face-to-face PDT. Internet-based treatment for adolescent depression has been found effective when based on both ICBT (7, 8) and IPDT (9). However, little is known about the mechanisms of change in these treatments.

It has been suggested that emotion regulation deficits may underlie almost all psychiatric disorders (10, 11). Numerous studies have linked maladaptive emotion regulation skills to psychopathology across many different disorders (12), with evidence derived from both cross-sectional and longitudinal studies. Experimental studies have repeatedly shown that maladaptive emotion regulation strategies impair recovery from negative affect/mood (13–15).

In a longitudinal study, a lower capacity for emotion regulation predicted higher depressive symptomatology over five years (16). A recent meta-analysis (17) found that remitted and currently depressed research participants reported maladaptive emotion regulation strategies to a much higher extent when compared to healthy controls. Fortunately, research suggests that emotion regulation problems are not characterized by deficits in strategies per se. Depressed patients are able to use more adaptive emotion regulation skills if instructed to do so. Hence, the problem seems to be maladaptive selection of emotion regulation strategies rather than a lack of capacity (18).

Compared to younger children, adolescents are experiencing a process of individuation and rely more on internal emotion regulation strategies, rather than being regulated by parents (19, 20). A combination of rapid development and increased social and emotional demands makes adolescence a time of heightened emotional intensity, reactivity, and fluctuations. This means that adolescence could be considered a time that places extra demands on emotion regulation (21).

A current body of evidence suggests links between disruptions in emotion regulation and psychopathology in adolescents, even if results are mixed [for a review, see Young et al. (21)]. A meta-analysis (22) found that emotion regulation strategies had negative associations with anxiety and depression, while maladaptive emotion regulation had positive associations. Recently, Gonçalves et al. (23) determined that difficulties in emotion regulation in early adolescence predicted depressive symptoms both cross-sectionally and over time. Thus, it can be suggested that increasing the capacity of adaptive emotion regulation might be an important focus for both preventive and therapeutic interventions (e.g. 22, 24, 25).

There are several studies on the topic of emotion regulation and psychological treatment. Emotion regulation group therapy has been found to reduce dysregulated emotions in adults (26). This approach has also been tested as an internet-based treatment targeting adolescents suffering from deliberate self-harm tendencies with promising results regarding improvement in emotion regulation (27). Suveg et al. (28) found that cognitive behavioral therapy (CBT) had limited effects on emotion dysregulation in the treatment of anxious youths. Emotion dysregulation decreased in relation to worry, but not in relation to feelings of anger or sadness. Recently, an internet-based psychodynamic treatment targeting adolescent depression resulted in large improvements (*d* = 0.92) in emotion dysregulation compared to a control condition (9). Several trials have also investigated emotion regulation as a predictor of outcome for CBT, rendering mixed results. Siegle, Carter, and Thase (29) found that depressed patients who exhibit deficits in emotion regulation may benefit more from CBT. This finding was corroborated by Niles et al. (30) for patients treated for social anxiety disorder. However, Nielsen et al. (31) found no evidence that emotion regulation at baseline predicted the rate of change among patients receiving group-delivered CBT for anxiety.

Several studies have found links between increased capacity for emotion regulation and improvement in depressive symptoms across treatments. Sauer-Zavala et al. (32) determined (using the Unified Protocol for the treatment for mixed emotional disorders) that changes in frequency of and reactivity to negative emotions, as well as awareness and acceptance of emotions, were related to change in depression. Increased capacity for emotion regulation predicted reductions in depressive symptoms through Affect Regulation Training (33) and CBT group treatment (34). Increased capacity for emotion regulation also had a mediating effect in an internet-based intervention for adolescents suffering from non-suicidal self-injury (27)

Even though the majority of research on the role of emotion regulation in psychological treatment has been conducted on treatments stemming from a CBT framework, emotion regulation has a prominent role in psychodynamic theory as well. The experiential‐dynamic emotion‐regulation model is grounded in both psychodynamic theory and affective neuroscience. It postulates that difficulties in emotion regulation either stem from dysregulated anxiety that has become a conditioned response to primary, adaptive feelings, and/or secondary, maladaptive emotions, which are products from defensive maneuvers (i.e. experiential avoidance) (35). According to psychodynamic theory, adaptive feelings can become conditioned to anxiety (fear of emotions) when they have been perceived as threatening to attachment relationships (36). This can happen when emotional expressions are dismissed, met with anger or leads to detachment from the significant other. These emotions will then be avoided through defensive maneuvers. Defenses solve the acute problem by repressing the emotions. However, in the long run they inhibit more adaptive ways of regulating and expressing one’s emotions and may therefore create and perpetuate symptoms of depression and/or anxiety (37).

To the best of our knowledge, no prior studies have investigated emotion regulation as a time-invariant or time-varying predictor of change in psychodynamic treatments. Thus, the primary aim of the present study was to investigate whether entry levels of emotion regulation deficits would predict the rate of change in the treatment of adolescent depression. A second aim was to evaluate whether changes in emotion regulation during treatment could predict future change in depression using a time lagged model.

## Method

### Setting

Data from the present study were collected in the ERiCA-project, which evaluated internet-based treatment for adolescent depression and was conducted by Stockholm University in close collaboration with Linköping University. The trial was approved by the Regional Ethics Board of Stockholm, Sweden (number: 2018/2268-31/5). Participants submitted written informed consent via the online treatment platform and received treatment at no cost. The International Standard Randomized Controlled Trial Number (ISRCTN) registration ID is 16206254.

### Recruitment and Participants

Recruitment took place during January and February of 2019. Participants were recruited through social media, schools, youth centers, and from youth mental health care providers. Participants were required to be between 15 and 18 years of age, and had to fulfill a diagnosis of MDD according to the DSM-5 (38). This diagnosis resulted from an assessment using the MINI International Neuropsychiatric Interview [MINI 7.0 (39)] and by scoring ≥10 points on the Quick Inventory of Depressive Symptomatology—Adolescent Self-Rated Version [QIDS-A17-SR (40)]. The diagnosis of MDD had to be the primary diagnosis. Exclusion criteria included prior suicide attempts and/or substantial suicidality (i.e. intent and/or plans to commit suicide expressed during intake interview or on screening forms), ongoing psychotropic medication that was not stable ≥ 3 months, and partaking in other psychological treatments. Furthermore, participants fulfilling any of the following diagnoses were also excluded: any psychotic disorder, bipolar I/II disorder, antisocial personality disorder, autism-spectrum disorder, or any substance use disorder. In the original trial, 76 adolescents were randomized, of whom 4 never entered treatment after randomization and 5 never entered treatment after being in the control group. Accordingly, the sample in the present study (n = 67) only consisted of adolescents entering treatment, including participants who were crossed over to treatment after the initial allocation to the control group. Patients’ demographics are presented in **Table 1**.

**Table 1:** Demographic data^a^.

	IPDT (n = 67)	
	n/M	%/SD
Female	55	82.1
Gender identity uncertain/other	3	4.5
Age	16.63	1.10
Major depressive disorder^b^	67	100
Any anxiety disorder^b^	40	59.7
PTSD^bc^	4	6.0
Eating disorder^bd^	3	4.5
Antidepressant medication	4	6.0
QIDS-A17-SR pretreatment	14.56	4.37

IPDT, Internet-based psychodynamic therapy; QIDS-A17-SR, quick inventory of depressive symptomatology adolescent version. ^a^Diagnostic assessment was conducted at baseline for the main RCT, ^b^Confirmed by the MINI-International Neuropsychiatric Interview. ^c^Posttraumatic stress syndrome. ^c^Bulimia nervosa/Binge-eating disorder.

### Instruments

Eligible participants were contacted via phone to conduct the MINI 7.0 (39) to establish psychiatric diagnoses. The MINI 7.0 was slightly altered by adding the irritability ﻿criterion to the depression module as well as the separation anxiety module from the MINI for Children and Adolescents (MINI KID). We also replaced the section assessing suicidality with the Columbia-Suicide Severity Rating Scale [C-SSRS (41)]. The C-SSRS was chosen because it is more easily administered and is recommended for use in clinical trials by the United States Food and Drug Administration (42). The MINI and C-SSRS were conducted by experienced registered clinical psychologists (n = 3) from the research team, in addition to students from the clinical psychology master’s program (n = 3) who received a full day of training in both instruments.

In the present study, two self-rated instruments were used for the analysis. Both were administered weekly as well as pre- and post-treatment via a secure internet platform. The primary outcome measure was QIDS-A17-SR, a reliable measure of depressive symptoms that has been found valid for both adult and adolescent populations (40, 43). Lindqvist et al. (9) reported an average Cronbach’s alpha across all time points of α = .76 (range:.71–.85), suggesting acceptable internal consistency in the present sample. The Difficulties in Emotion Regulation Scale [DERS-16 (44)] was used as a measure of the capacity for emotion regulation. The original 36-item version of DERS is a comprehensive measure of emotion dysregulation encompassing six distinct (although related) dimensions of emotion regulation. These dimensions are lack of awareness, clarity of emotions, difficulties in controlling impulsive behavior, non-acceptance of emotions, engaging in goal-directed behaviors when distressed, and limited access to emotion regulation techniques that are perceived as effective (45). The DERS-16 is a short form, developed from the original DERS, measuring overall capacity for emotion regulation. Items from all subscales (except for lack of emotional awareness) have been retained in DERS-16. It should be noted that analyses have suggested only minor differences in convergent and discriminant validity between the scales (44). For the present study, DERS-16 was chosen over the original scale as its brevity allows for weekly measurement of emotion regulation. Lindqvist et al. (9) reported good internal consistency (α = .89) in the present sample.

### Intervention

The IPDT was eight weeks long and consisted of eight weekly-administered self-help modules consisting of texts, videos, and exercises (9). Exercises were reported on the platform and all participants received weekly messages with feedback from their therapist. To reduce attrition and increase motivation, the intervention also contained a weekly 30-minute synchronous text chat session between therapist and participant. Due to limited resources, only the first group of patients (n = 34) received chat sessions. The remainder (n = 33) received the same treatment but without additional synchronous chat sessions. All communications between participants and their therapists were conducted through an encrypted online platform (46).

The treatment presents the possibility of inner, emotional conflicts triggering and maintaining symptoms of depression. The participants are introduced to theory about emotions and how and why they can be repressed with defenses. Through the treatment program participants learn to differentiate between different bodily symptoms of anxiety and how to regulate emotions and anxiety without using maladaptive defenses. This is done by enhancing their capacity for self-observation through acquired bodily awareness and by learning to observe their own emotional reactions, especially in relation to others. Furthermore, participants are encouraged to experience and express emotions that have been previously warded off. This gradual exposure will lead to the emotions being uncoupled from anxiety, and the use of maladaptive emotion regulation strategies (i.e. defenses) will no longer be needed.

### Statistical Analysis

The study used all available data on participants entering treatment (n = 67). The original outcome study (9) presents the main findings from the RCT. Data in the present study were analyzed based on all patients entering treatment, regardless of whether they dropped out (i.e. intent to treat). Growth curves were estimated using all available data. Model building started with estimating a basic time model including random intercepts and slopes for time. To account for possible non-linearity in the data a quadratic term (TIME × TIME) and a cubic term (TIME × TIME×TIME) for time were tested and discarded as neither reached significance or improved model fit. All models were analyzed with full maximum likelihood and all statistical analyses were conducted using SPSS v.26 (IBM Corp., Armonk, NY).

#### DERS-16 as a Time-Invariant Predictor

In this model, we analyzed whether pretreatment difficulties in emotion regulation predict within-group changes in the outcome variable during treatment. Within-group effect sizes were calculated using model estimated differences in pre- and postmeans and the observed pretreatment standard deviation, as recommended by Feingold (47).

A series of multilevel models of the trajectories of the QIDS-A17-SR were tested. First, an unconditional growth model was estimated to examine the average growth over time in treatment, represented by the following equations:

Level 1:

$$QIDS–A17–SR_{it}=\beta_{0i}+\beta_{1i}\left(TIME_{t}\right)+\epsilon_{it}$$

Here, $QIDS–A17–SR_{it}$represents the depression score for individual *i* at time *t*; $\beta_{0i}$ represents the intercept for individual *i* at time 0 (i.e. at baseline); $\beta_{1i}$ represents the linear rate of growth for individual *i* across each time point; and $\epsilon_{it}$is the error term indicating the deviation of individual *i’s* score from their own estimated regression line at each time point *(t)*.

Level 2:

$$\beta_{0i}=\gamma_{00}+u_{0i}$$

$$\beta_{1i}=\gamma_{10}+u_{1i}$$

Here, each individual’s intercept, β_0*i*_, is modeled as the grand mean of all individuals’ scores at Time 0 ($\gamma_{00}$), plus each individual’s deviation from that grand mean at time 0 ($u_{0i}$). Term β *_1i_* represents the linear rate of growth across all time points for each individual, γ *_10_*represents the average rate of change for all individuals across all time points, and *u_1i_* represents each individual’s growth parameter deviation from that average.

Next, a conditional growth model was estimated to examine whether between-person differences in change over time were affected by initial levels of emotion dysregulation (DERS-16). Thus, equations on Level 2 are changed accordingly for the conditional model (while the equation on Level 1 is identical to the previous equation):

$$\beta_{0i}=\gamma_{00}+\gamma_{01}\left(DERS-16\right)+u_{0i}$$

$$\beta_{1i}=\gamma_{10}+\gamma_{11}\left(DERS-16\right)+u_{1i}$$

To assess the effect of DERS-16 scores on the individual intercept, $\gamma_{00}$ is the grand mean at time 0, $\gamma_{01}$ is the contribution of DERS-16 to the intercept value, and $u_{0i}$ represents each individual’s deviation from the modeled intercept value. The second equation estimates the extent to which DERS-16 scores affect the rate of change in QIDS-A17-SR. Term $\gamma_{10}$ represents the average rate of change for all individuals across all time points, $\gamma_{11}$ depicts the influence of DERS-16 scores on the rate of change, and $u_{1i}$ represents each individual’s growth parameter deviation from the estimated slope. Level 1 residuals were assumed independent and identically distributed. At Level 2, we used an unstructured covariance structure, allowing intercept and slope to correlate. As a post-hoc test, to control for potential confounders, gender and a centered variable measuring adherence to the treatment program (i.e. number of modules opened) were added in interaction with Time.

#### DERS-16 as a Time-Varying Predictor in IPDT

To investigate whether within-person change in DERS-16 predicted change in depression the following week, we employed linear mixed effects modeling to analyze individual change over time (48). The time-lagged effects of DERS-16 scores on depression were analyzed as follows: the effect of DERS-16 the week before (time point t −1) was used to predict QIDS-A17-SR the following week (time point t). Patients providing at least one complete data point for both DERS-16 and the subsequent QIDS-A17-SR contributed to the models.

To separate within- and between-person effects, DERS-16 was divided into two predictors. The first predictor was a time-invariant variable consisting of the individual’s total mean of all DERS-16 scores across all time points, from which the grand mean of all individual’s DERS-16 scores during the entire treatment was subtracted. In other words, this predictor illustrates the difference between individuals on the pooled DERS-16 scores across all assessment points (i.e. the grand mean centered between-person effect on DERS-16). The second predictor was calculated by subtracting each individual’s mean DERS-16 value over all time points from their DERS-16 value at each time point. This within predictor, a so-called time-varying person-mean centered variable, was calculated to account for the within-person effect of DERS-16 scores on depression.

As a final step in the analysis, we included fixed and random effects of time in the model to control for general effects of time. Due to the autoregressive nature of the time series data, we used the first order autoregressive (AR[1]) structure for the within-person residuals in all analyses. On the between-person level, an unstructured covariance structure was chosen, which allowed intercept and slopes to correlate. The final analysis is illustrated using the following equation:

Level 1

$$QIDS–A17–SR_{it}=\beta_{00i}+\beta_{10}\left(DERS–16_{it–1}-{\bar{DERS-16}}_{i}\right)+\beta_{20i}\left(TIME_{t}\right)+\epsilon_{it}$$

Here, $QIDS–A17–SR_{it}$ is the individual’s depression score at time point t. The equation also illustrates fixed effects on intercept ($\beta_{00i}$) and the fixed within-person effect of DERS-16 on QIDS-A17-SR the following week, i.e. the time lagged effect of person-mean centered DERS-16 [$\beta_{10}\left(DERS–16_{it–1}-{\bar{DERS-16}}_{i}\right)$]. Furthermore, this includes the fixed effect of time $\beta_{20i}$ on QIDS-A17-SR and ϵ*_it_* represents the deviation of individual’s *(i)* score from their own modeled line at each time point *(t)*.

Level 2

$$\beta_{00i}=\gamma_{00}+\gamma_{01}\left({\bar{DERS–16}}_{i}-\bar{\text{DERS}–16}\right)+u_{0i}$$

$$\beta_{10}=\gamma_{10}$$

$$\beta_{20i}=\gamma_{20}+u_{1i}$$

Level 2 also includes random intercepts ($\beta_{00i}$), as illustrated in the first equation. Here, $\gamma_{00}$ is the grand mean at time 0, $\gamma_{01}\left({\bar{DERS–16}}_{i}-\bar{\text{DERS}–16}\right)$ is the between-person (grand mean centered) effect of DERS-16 on the intercept value, and *u_0i_* represents each individual’s deviation from the modeled intercept value. $\;\gamma_{10}$ represents the fixed, time lagged effect of person-mean centered DERS-16.

This level also contains random slopes, as illustrated in the third equation. Here, $\beta_{20i}\;$ represents the linear rate of growth across all time points for each individual, $\gamma_{20}$ represents the average rate of change for all individuals across all time points and $u_{1i}\;$ represents each individual’s growth parameter deviation from that average. As a post-hoc test, to control for potential confounders, gender and a centered variable measuring adherence to the treatment program (i.e. number of modules opened) were added in interaction with the lagged DERS-16.

#### The Effect Sizes of DERS-16 as a Time-Invariant and Time-Varying Predictor

To make the results more easily interpretable, we also estimated pseudo-R^2^, i.e. the proportion of residual variance explained by the within-person changes in DERS-16 (48). For DERS-16 as a time-varying predictor, pseudo-R^2^ was calculated by dividing the difference in residual variance between models (with and without the within-person effects of DERS-16) by the residual variance from the model without the within-person predictor. However, in the calculations of DERS-16 as a time-invariant predictor, the residual variance actually increased when adding the predictor, resulting in negative explained variance. While this is a known phenomenon (49), it basically renders pseudo-R^2^ uninterpretable; hence, we choose not to present the result.

## Results

### Adherence and Attrition

The average participant opened 6.6 treatment modules (SD = 2.31), 64.2% (n = 43) opened all modules, 83.6% (n = 56) opened more than half, and 6% (n = 4) terminated treatment prematurely. **Table 2** presents observed values for QIDS-A17-SR and DERS-16 across treatment.

**Table 2:** Observed means, standard deviations, and number of observations for outcome and processes over the treatment period.

Measure	Week									
	0	1	2	3	4	5	6	7	8	9
QIDS-A17-SR										
*M*	14.40	14.28	13.55	12.89	12.13	11.78	11.94	11.60	10.59	9.46
*SD*	4.52	4.08	4.6	4.52	4.14	4.20	4.31	4.60	5.02	4.98
*n*	67	67	65	57	55	55	50	55	46	59
DERS-16										
*M*	55.3	54.55	55.14	53.18	51.33	50.02	48.40	45.73	44.82	41.02
*SD*	11.73	12.55	12.13	11.66	11.84	12.26	11.62	12.27	13.86	14.21
*n*	67	67	65	57	55	55	50	55	45	57

QIDS-A17-SR, quick inventory of depressive symptomatology adolescent self-rated version; DERS-16, difficulties in emotion regulation scale brief version.

### Effects of Treatment

Results from the unconditional growth model for QIDS-A17-SR indicated that there was significant variance in the intercept (symptom level at baseline: 13.01, *p* <.001) and in the slope (rate of decrease in QIDS-A17-SR scores over time: 0.15, *p* <.001). This significant variance implies it might be worthwhile investigating possible predictors of change to further our understanding of treatment effects. The mean QIDS-A17-SR trajectory was estimated to start at 14.42 (at baseline) and the estimated average decrease was −0.46 per week in treatment. The effect size (Cohen’s *d*), pre to post, for the treatment was 0.92, 95% CI [0.68, 1.16]. The correlation between intercept and slope was −0.16, indicating that patients starting with higher baseline scores on QIDS-A17-SR experienced a steeper decline of depressive symptomatology. While the correlation was statistically non-significant (*p* = .559), we chose to retain the unstructured covariance structure to control for regression to the mean in the conditional growth model.

### Effects of Pretreatment Difficulties in Emotion Regulation on Outcome

In a conditional growth model, DERS-16 was added at Level 2 as a time-invariant predictor in interaction with intercept and with time. The DERS−16 × TIME interaction was statistically significant (*p* = .041), indicating that relatively higher pretreatment DERS-16 scores significantly predicted increased growth rate in QIDS-A17-SR during treatment. Adding Gender in interaction with time did not reach statistical significance (*p = .*88) and was dropped from the analysis. Adding centered adherence (modules opened) in interaction with time did reach statistical significance (*p* = .04), indicating that participants taking part of more of the treatment material had larger effects. However, this did not affect the effects of pretreatment scores on DERS-16 on QIDS-A17-SR growth rates and was thus dropped from the analysis.

**Figure 1:**
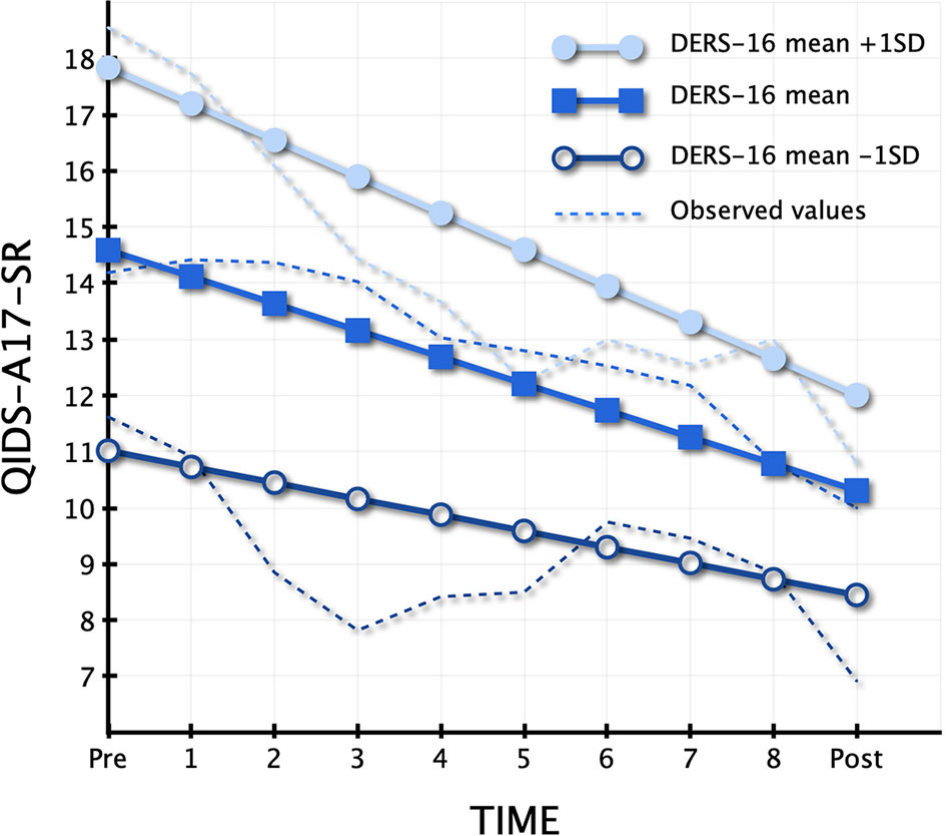
Different trajectories of change depending on baseline value on Difficulties in Emotion Regulation Scale (DERS-16).

For more numerical details, see **Table 3**. **Figure 1** illustrates the different rates of growth in depression in patients presenting relatively high (+1 SD), average and relatively low scores (−1 SD) in the present sample on DERS-16.

**Table 3:** Effects of baseline scores of DERS-16 on rate of change in QIDS-A17-SR: parameter estimates, standard errors, 95% confidence intervals, and p-values (n = 67).

Model estimates		Estimate *(SE)*	95% CI	*P* value
**Fixed effects**				
	γ*_00_* (model intercept)	14.38 (0.38)	13.60, 15.15	<.001
	γ*_01_*(effect of grand mean centered DERS-16 on model intercept)	0.20 (0.03)	0.13, 0.27	<.001
	γ*_10_ (effects of time on outcome)*	–0.46 (0.06)	–0.58, –0.36	<.001
	γ*_11_*(effect of DERS-16 on rate of change)	–0.01(0.00)	–0.02, –0.00	.041
**Random effects**				
	u_0i_ (variance intercept)	7.79 (1.71)	4.97, 12.21	<.001
	u_1i_ (variance slopes)	0.14 (0.04)	0.08, 0.25	<.001
	Correlation between intercept and slope	–0.16 (0.21)	–0.25, 0.52	.45
	ϵ*_it_* (Residual variance)	6.07 (0.41)	5.32, 6.93	<.001

QIDS-A17-SR, quick inventory of depressive symptomatology adolescent self-rated version; DERS-16, difficulties in emotion regulation scale brief version.

### Effects of Intraindividual Changes in Difficulties in Emotion Regulation on Outcome

Results from linear mixed effects models, examining within-person effects of DERS-16 on QIDS-A17-SR the following week, are presented in **Table 4**. The within-person effects of DERS-16 were significant through all analyses, even when controlling for effects of time (i.e. detrending). The time-lagged relationship was in the expected direction, meaning that lower scores on DERS-16 predicted lower scores on QIDS-A17-SR the subsequent week.

**Table 4:** Within-person effects of DERS-16 on QIDS-A17-SR: parameter estimates, standard errors, 95% confidence intervals, and p values (n = 67).

	DERS-16 on QIDS-A17-SR (without controlling for time)			DERS-16 on QIDS-A17-SR (when controlling for time)		
	Estimates (*SE*)	95% CI	p-value	Estimates (*SE*)	95% CI	p-value
**Fixed effects**						
*γ*_00_ (model intercept)	12.15 (0.39)	11.38, 12.93	<.001	13.90 (0.39)	13.12, 14.68	<.001
*γ*_10_ (within-person effect, lagged process on outcome)	0.11 (0.02)	0.07, 0.14	<.001	0.07 (0.02)	0.03, 0.10	<.001
*γ*_20_ (effect of time on outcome)	N/A	N/A	N/A	−0.39 (0.06)	−0.52, −0.27	<.001
*γ*_01_ (between-person effect on outcome)	0.26 (0.04)	0.18, 0.33	<.001	0.25 (0.03)	0.18, 0.32	<.001
**Random effects**						
u_0i_ (variance intercept)	7.64 (0.67)	6.43, 9.07	<.001	4.94 (1.78)	2.44, 10.00	<.01
u_2i_ (variance slopes for time)	N/A	N/A	N/A	0.06 (0.05)	0.01, 0.29	.24
Correlation intercept and slopes	N/A	N/A	N/A	0.68 (0.66)	−0.91, 1.00	.30
ϵ*_it_* (residual variance)	8.29			6.19		
*Pseudo-R* ^2^ (explained variance)	41.23%			8.70%		

Note. QIDS-A17-SR, quick inventory of depressive symptomatology adolescent self-rated; DERS-16, difficulties in emotion regulation scale brief version.

First, we estimated a model with fixed between- and within-person effects of DERS-16 without controlling for the effect of time. This model estimated a significant effect of within-person DERS-16, where a 1-point reduction in DERS-16 predicted a reduction of 0.11 points in QIDS-A17-SR the following week (γ_10_ = 0.11; SE = 0.02; 95% CI [0.07 to 0.14]). Using the formula for pseudo-R^2^, the added within-person effects of DERS-16 explained 41.23% of the variance in QIDS-A-17 the following week, which implies that approximately 41% of the change in QIDS-A17-SR stems from within-person changes in DERS-16.

In the final detrended model, controlling for the effect of time, the estimate of the time-lagged within-person DERS-16 was substantially lower, which was as expected. The parameter estimate for the within-person effect of DERS-16 indicates that a reduction of 1 point in DERS-16 predicted a reduction of 0.07 points in QIDS-A17-SR the following week. To illustrate this effect and make it more interpretable, we calculated pseudo-R^2^: 8.70% of the variance in QIDS-A17-SR could be attributed to within-person changes of DERS-16.

Adding gender in interaction with lagged DERS-16 did not reach significance (*p* = .35) and it was therefore dropped. However, centered treatment adherence (number of modules opened) in interaction with the lagged DERS-16 did reach significance (*p* = .005), indicating that for each module opened during the entirety of the treatment, the effect of lagged DERS-16 on the following week QIDS-A17-SR increased by 0.03 points.

## Discussion

The aim of the present study was to evaluate the role of emotion regulation, as measured by DERS-16, in IPDT for adolescent depression. Emotion regulation was investigated as both a time-invariant and time-varying predictor of change in depression. Our findings suggest that emotion regulation indeed plays an important role in the treatment of adolescent depression with IPDT. Emotion regulation measured at baseline affected the rate of change in depression, where patients exhibiting more dysregulated affects made somewhat larger gains from treatment. Furthermore, our analyses imply that an increased capacity for emotion regulation might act as a mechanism of change in IPDT as within-person changes in emotion regulation during treatment predicted change in depression the following week.

The findings on emotion regulation as a baseline predictor imply that patients entering treatment with relatively larger deficits in emotion regulation benefit somewhat more from IPDT than patients with relatively less dysregulated emotions. Our results are in agreement with prior studies conducted on CBT (29, 30), suggesting that larger deficits in emotion regulation predict better treatment responses.

Our findings that intraindividual changes in emotion regulation predict outcome are in agreement with research on emotion-focused CBT (32), traditional CBT (34), and Affect Regulation Training (33), where increased emotion regulation has been shown to act as a potential mechanism of change. In fact, results from Berking et al. (33) suggest that increased capacity for emotion regulation might act as a common mechanism of change for treatments targeting depression, albeit with different theoretical underpinnings. This finding is corroborated by the present study.

The present study elucidates the process of change in the treatment of adolescent depression and presents an increased capacity for emotion regulation as a possible mechanism of change in IPDT. This raises the question whether this is also the case in experiential dynamic therapies (EDTs) in general. The model of psychopathology in IPDT is clearly based on psychodynamic principles (9). The treatment relies heavily on working with dysregulating defenses (i.e. unconscious strategies leading to experiential avoidance), while also targeting dysregulated anxiety through an increased capacity for self-observation and bodily awareness. These aims are often described as core elements in EDTs (50). This focus might be a possible explanation for our finding that greater difficulties in emotion regulation at baseline predicted treatment response. The treatment might target problems that are more relevant to young persons with relatively more severe emotion regulation problems, whilst depressed adolescents with less problems with emotion regulation would be helped more by interventions targeting other difficulties. Targeting emotion regulation might be particularly important in the treatment of young people as they are in a gradual process of learning to rely more on internal emotion regulation strategies, rather than depending on being regulated by significant others (19, 20). This is an area for future research. Further studies should also investigate if these results also apply to face-to-face PDT in the treatment of adolescent depression.

Another reason to look into further possible mechanisms of change in IPDT is the fact that increased intraindividual capacity for emotion regulation only predicted roughly 9% of variance in the outcome (after detrending, i.e. controlling for general effects of time). This implies there are probably several mechanisms of change yet to be empirically tested. Further studies on IPDT should investigate other theoretically sound concepts (i.e. mentalization, insight, and self-compassion) as mechanisms of change. Furthermore, future research should focus on investigating whether it is treatment components, common factors, or a combination of both that facilitate change in emotion regulation.

Because more difficulties in emotion regulation correlated with more severe depression, this could raise concerns about whether the extent of the effect of baseline emotion regulation on rate of change in depressive symptoms could merely be an effect of regression to the mean. However, by allowing intercept and slope to correlate, we controlled for the possible effect of regression to the mean.

Detrending of data to control for time is a debated subject in the context of analyses of within-person effects. On the one hand, this is an experimental study where we assume that changes in both emotion regulation and depression are caused by our experimental manipulation, i.e. the treatment. Detrending removes the effect of treatment, meaning that we actually risk removing at least some of the effects we intended to study (51). On the other hand, since the current study only presents results for participants in treatment and not a control group, it could be argued that both effects are to some extent unrelated to treatment but caused merely by the passage of time (i.e. spontaneous remission). In this case, detrending for time would be necessary. Lindqvist et al. (9) showed that the treatment had large effects on both depressive symptoms and emotion regulation compared to a control condition (*d* = 0.82 and *d* = 0.97, respectively), rendering this explanation unlikely. However, one cannot be completely certain that there are no confounders associated with the time-trends in the data (52). This is why we chose to present results both with and without detrending. To explain this in relation to our results, the effect of intraindividual changes in DERS-16 remained significant (even when detrending for time), which strengthens claims for causality. However, it is also possible that the detrending leads to an underestimation of the effects of emotion regulation on depression.

### Strengths and Limitations

An apparent strength of the study is the multilevel framework, enabling us to separate within-and between-person variances, meaning that we could investigate effects on both the within- and between-person levels as well as make use of all available data. Weekly measurements of both predictor and outcome variables allowed us to investigate relationships between emotion regulation and depression over time in treatment, strengthening claims of causality.

One limitation of the study is the lack of a control group and random allocation. It could be argued that this prevents us from attributing the change process to the actual treatment. The significant, time-lagged and detrended results strengthens claims of causality, but it is still possible that confounders influenced our results and that this effect would have been seen in any remission from depression regardless of treatment. On the other hand, the treatment material and study therapists address many of the causes, as assumed in EDT, underlying emotion dysregulation (50). Furthermore, the post-hoc analysis indicating that participants who read more of the material had larger effects of emotion regulation on depression the following week strengthens the proposed pathway of treatment enhancing capacity for emotion regulation. Further research should be done comparing the processes in IPDT to control conditions and/or different internet-based treatments and their respective impact on depression through enhanced emotion regulation.

A higher frequency of assessments during treatment could have furthered our knowledge about the temporal relationships between emotion regulation and depression. In addition, the study only included one time-varying predictor measured weekly during treatment.

A final limitation worth mentioning is the relatively small sample size, possibly limiting the generalizability of the findings as well as making the estimates less precise. The present study should be replicated to establish increased emotion regulation as a mechanism of change in IPDT. As the present study investigates the role of emotion regulation in IPDT it is unclear to what extent the results can be generalized to the psychotherapeutic process in face-to-face PDTs.

### Conclusions

The results of the present study highlight the importance of emotion regulation as both a time-invariant and time-varying predictor of change in symptoms of depression in IPDT. The results imply that depressed patients expressing relatively higher degrees of dysregulated affect at intake experience larger treatment effects in IPDT. Also, an improved capacity for emotion regulation, presumably acquired through treatment, precedes improvement in depression. The effect of intraindividual changes in emotion regulation is in accordance with theory in EDTs. This theory postulates that recognizing and relinquishing defenses and regulating anxiety should lead to less dysregulated affective states and greater access to underlying adaptive emotions which in turn leads to symptom reduction. Further studies are needed to confirm these results, preferably on both IPDT and PDT delivered face-to-face.

## Data Availability Statement

The datasets presented in this article are not readily available because participants are mostly minors and it contains sensitive data. Therefore, the dataset is available on reasonable requests as deemed by the principal investigator of the study. Requests to access the datasets should be directed to BP, bjorn.philips@psychology.su.se.

## Ethics Statement

The studies involving human participants were reviewed and approved by the Regional Ethics Board of Stockholm, Sweden (number: 2018/2268-31/5). Written informed consent from the participants’ legal guardian/next of kin was not required to participate in this study in accordance with the national legislation and the institutional requirements.

## Author Contributions

All authors contributed to the article and approved the submitted version. JM, KL, and FF conducted the statistical analysis. JM and KL drafted the first version of the text while FF, GA, BP, and PC provided feedback and reviewed and revised the manuscript.

## Funding

This work was supported by the Kavli Trust under grant number 32/18.

## Conflict of Interest

The authors declare that the research was conducted in the absence of any commercial or financial relationships that could be construed as a potential conflict of interest.
